# The association between alcohol and tobacco use among elementary and high school students in Crete, Greece

**DOI:** 10.1186/1617-9625-10-15

**Published:** 2012-09-25

**Authors:** Ioanna G Tsiligianni, Constantine Ilias Vardavas, Izolde Bouloukaki, Epameinondas Kosmas, Evgenia Verigou, Maria Kiriakaki, Nikolaos Siafakas, Nikolaos Tzanakis

**Affiliations:** 1 Department of Thoracic Medicine, School of Medicine, University of Crete, Heraklion, Crete, P.O 71003, Greece; 2 Department of Social Medicine, School of Medicine, University of Crete, Heraklion, Crete, P.O 71003, Greece; 3 Agia Barbara Health Care Center, Heraklion, Crete, P.O 70003, Greece; 4 3rd Department of Pulmonary Medicine, Chest Diseases Hospital "Sotiria", Athens, P.O 11527, Greece; 5 Prefecture of Lasithi, Agios Nikolaos, Crete, P.O. 72100, Greece

## Abstract

**Background:**

Tobacco and alcohol use during adolescence have potential long term health consequences and a possibility of future addiction.

**Methods:**

This cross sectional study took place in 2007 among a convenience sample of 981 adolescents from public elementary and high schools in Eastern Crete, Greece. Following parental consent, an anonymous structured questionnaire including information on personal and family use of alcohol and tobacco was distributed.

**Results:**

Among the entire study population, cigarette experimentation was found to be associated with current alcohol use, with an Adjusted Odds Ratio (aOR) of 38.8; (95%C.I: 5.33-58.2) and with having a smoker in the immediate family (aOR 10.3; 95%C.I: 3.14-34.0). Among the subset of elementary school children, cigarette smoking was strongly associated with current alcohol use aOR 9.7; (95%C.I: 2.12-44.3), while the association between smoking experimentation and sibling and parental alcohol use was statistically significant within the entire population (however not among elementary students) with an aOR of 2.76 (95%C.I: 1.24-6.15) and aOR 3.66, (95%C.I: 1.97-6.81) respectively. The elementary child’s gender was not found to be associated with cigarette experimentation among this study population.

**Conclusions:**

Strong associations were found between alcohol use and tobacco experimentation. The potential parental influence on consequent adolescent tobacco and alcohol use was also noted. Potential community based interventions, if launched in Greece, should take the role of the Greek family into account.

## Introduction

Smoking is a leading cause of preventable disease and disability [1]. Research has indicated that there is a strong tendency for cigarette smoking and alcohol dependence to co-occur, while evidence from a number of studies has indicated that cigarette smoking during adolescence is a strong determinant of subsequent alcohol use and abuse, with a younger age of experimentation with cigarettes associated with higher dependency rates during adult life [2-4].

Within Greece, both smoking and alcohol use have been highlighted as areas of public health concern with previously estimated smoking rates found to range between 10-50%, depending on the age group, year and place of survey [5-9]. According to the most recent Global Youth Tobacco Survey (GYTS), which was implemented in 2005 among a representative sample of 13–15 year olds in Greece, one third reported that they had tried tobacco in the past, while 16% reported themselves as current users of tobacco products [5]. Additionally, alcohol use has also been reported to be elevated, although on a decreasing trend [10].

With the above in mind, the investigation of factors associated with the potential clustering of behavioural risk factors among youth could help define the future burden of disease and identify targets for prevention programs [11]. Acknowledging that tobacco sales to minors was allowed at the time of the study (tobacco sales to minors was subsequently banned in September 2009), within the present study we aimed to investigate the relationship between cigarette experimentation and alcohol use among elementary and high school students in Eastern Crete, Greece.

## Methods

This cross sectional study took place during 2007, within the 5 major towns (Agios Nikolaos, Siteia, Ierapetra, Neapolis and Tzermiado) of the prefecture of Lassithi in Eastern Crete, Greece. A convenience sample of 981 adolescents participated in the study, 52.5% of which were female (n = 515). The majority of the participants were currently enrolled in elementary school (59.7%, n = 586) aged 9.4 ± 0.8 years; while 27.6% (n = 270), aged 12.7 ± 1.3 years; and 12.1% (n = 119), aged 16.1 ± 0.7 were enrolled in junior and senior high schools respectively. Ethics approval was provided by the Medical Ethics Committee of the University Hospital of Heraklion.

Following informed written parental consent, the participating school children were handed an anonymous structured questionnaire that covered the youth’s current tobacco and alcohol use, while demographic, and family related characteristics were also noted. Smoking experimentation was defined as “Have you ever experimented with cigarette smoking, even for a few puffs?”, while current smoking status was asked by using the question “Over the past month, have you smoked one or more cigarettes?” Similarly, regarding alcohol use, current alcohol use was defined as consuming any alcohol over the past month, while the age of first alcohol use was also noted. Similarly parental and sibling alcohol use was assessed through the question “Have you seen your parents/siblings drink alcohol over the past month?”, while excessive drinking was assessed through the question “How many times in the past have you had so much to drink that you felt ill?”. Although 981 questionnaires were returned for analyses, complete data was available for 977, which was our final study sample. In total, 462 boys (47.1%) and 515 girls provided completed questionnaires. The overall response rate was 97%.

All p-values are based on two-sided tests and a significance level lower than 0.05 was defined as significant. Continuous variables are presented as mean ± standard deviation, while qualitative variables were depicted with the use of frequencies. Chi-squared (*χ*^2^) tests were used for detecting differences between study groups in regards to their personal characteristics. Logistic regression analyses were also performed so as to assess the relationship between tobacco and alcohol use, during which two distinct analyses were performed, one for elementary school children and one for the entire study population. The statistical analysis was performed with the statistical package SPSS 19.0. (Statistical Package for Social Sciences, SPSS, Inc, Illinois, USA).

## Results

Cigarette experimentation was estimated at 8.2% (n = 48) among elementary school students, 25.2% (n = 30) among junior high school students and 42.5% (114) among senior high school students, with the current prevalence of smoking among our study population estimated at 3.8%, 17.6% and 18.9% respectively. Furthermore 27% of the study population had consumed alcohol in the past month, with 5% of elementary school children under the age of 10 already experimented with tobacco and 15% already experimented with alcohol. The characteristics of tobacco and alcohol use among our study sample, is depicted in Table 1.

**Table 1:** Characteristics of tobacco and alcohol use among elementary and high school students in Crete, Greece in 2007

**Characteristics of tobacco and alcohol use**	**Total**^**1**^**n (%)**
**Have you ever experimented with cigarettes, even one or more puffs?**	
No	770 (80.0)
Yes	193 (20.0)
**Do you currently smoke cigarettes?**	
Non smoker	886 (90.3)
Smoker	95 (9.7)
**Age at first tobacco experimentation (current smokers only)**	
<10	43 (22.4)
10-12	60 (31.2)
+12	89 (46.4)
**Family smoking status?**	
No smokers in the immediate family	279 (29.4)
Maternal smoking	91 (9.6)
Paternal smoking	298 (31.4)
Dual parental smoking	179 (18.9)
Sibling smoking	24 (2.5)
All members of family are smokers	78 (8.2)
**Have you had a glass of alcohol over the past month?**	
No	710 (72.4)
Yes	271 (27.6)
**Have you seen your parents drink alcohol over the past month?**	
No	828 (87.6)
Yes	117 (12.4)
**Have you seen your brother/sister drink alcohol over the past month?**	
No	873 (94.5)
Yes	47 (5.5)
**How many times in the past have you had so much to drink that you felt ill**	
Never	408 (69.7)
1-2	143 (24.4)
3+	37 (6.3)

^1^ Percentages are based on the number of respondents to each answer.

Within Table 2, we assessed the association between alcohol and tobacco use among both elementary and high school students. Notably, according to the bivariate analyses, current smokers were more likely to have a smoker in the immediate family, or parents/siblings that use alcohol (p < 0.001).

**Table 2:** Associations between alcohol and tobacco use among elementary and high school students in Crete, Greece, 2007

	**Alcohol consumers (n = 271)**	**Non-alcohol consumers (n = 710)**	**p- value**	**Current smokers (n = 95)**	**Current non-smokers (n = 886)**	**p- value**	**Current smokers & alcohol consumers**	**Current non-smokers & non-alcohol consumers**	**p- value**
	**n (%)**	**n (%)**		**n (%)**	**n (%)**		**(n = 60)**	**(n = 675)**	
**Gender**									
Male	149 (32.3)	313 (67.7)	**0.003**	53 (11.5)	409 (88.5)	0.08	35 (10.6)	295 (89.4)	**0.03**
Female	122 (23.7)	393 (76.3)		42 (8.2)	473 (91.8)		25 (6.2)	376 (93.8)	
**Class of schooling**									
Elementary school	68 (11.6)	518 (88.4)	**<0.001**	22 (3.8)	564 (96.2)	**<0.001**	11 (2.1)	507 (97.9)	**<0.001**
Junior high school	39 (32.8)	80 (67.2)		21 (17.6)	98 (82.4)		10 (12.7)	69 (87.3)	
Senior high school	161 (59.6)	109 (40.4)		51 (18.9)	219 (81.1)		38 (28.4)	96 (71.6)	
**Smoker in immediate family**									
No	55 (19.7)	224 (80.3)	**<0.001**	3 (1.1)	276 (98.9)	**<0.001**	3 (1.3)	224 (98.7)	0.40
Yes	207 (30.9)	463 (69.1)		91 (13.6)	579 (86.4)		57 (11.7)	429 (88.3)	
**Seen parents drink alcohol over the past month**									
No	206 (24.9)	622 (75.1)	**<0.001**	53 (6.4)	775 (93.6)	**<0.001**	35 (5.5)	604 (94.5)	**<0.001**
Yes	62 (53.0)	55 (47.0)		40 (34.2)	77 (65.8)		25 (38.5)	40 (61.5)	
**Seen siblings drink alcohol over the past month**									
No	242 (27.7)	631 (72.3)	**<0.001**	67 (7.7)	806 (92.3)	**<0.001**	44 (6.7)	608 (93.3)	**<0.001**
Yes	25 (53.2)	22 (46.8)		23 (48.9)	24 (51.1)		14 (51.9)	13 (48.1)	

Within the regression analysis of Table 3, smoking experimentation among elementary school students was strongly associated with current alcohol use (OR 9.7, 95%C.I: 2.12-44.3), while the association between smoking experimentation and parental alcohol use was borderline non-statistically significant (OR 3.03, 95%C.I: 0.99-9.23) for elementary students, and statistically significant for high school students (OR 3.66, 95%C.I: 1.97-6.81). The elementary school students’ gender and sibling alcohol use were not found to be associated with cigarette experimentation, while among high school students, only the later was statistically significant (OR 2.76; 95%C.I: 1.24-6.15). Finally, among the entire study population, cigarette experimentation was found to be associated with current alcohol use (OR 38.8, 95%C.I: 5.33-58.2) and with having a smoker in the immediate family (OR 10.3; 95%C.I: 3.14-34.0).

**Table 3:** **Association between cigarette experimentation, and personal/family tobacco and alcohol use among school students**^**2**^**in Crete, Greece, 2007**

	**Elementary school students**^**1**^		**Elementary and high school students**^**2**^	
	**Unadjusted**	**Adjusted**^**3**^	**Unadjusted**	**Adjusted**^**3**^
	**OR (95% C.I)**	**OR (95% C.I)**	**OR (95% C.I)**	**OR (95% C.I)**
Gender (males)	2.05 (0.82-5.09)	1.4 (0.49-3.94)	1.45 (0.65-2.23)	1.46 (0.89-2.40)
Smoker in immediate family^4^ (yes)	--	--	14.5 (4.54-46.1)	10.3 (3.14-34.0)
Currently drink alcohol (yes)	5.95 (2.16-16.4)	9.71 (2.12-44.3)	13.8 (5.59-34.5)	38.8 (5.33-58.2)
Parents drink alcohol (yes)	6.00 (2.35-15.3)	3.03 (0.99-9.23)	7.59 (4.74-12.2)	3.66 (1.97-6.81)
Siblings drink alcohol (yes)	4.17 (0.88-19.8)	1.05 (0.18-6.06)	11.5 (6.18-21.5)	2.76 (1.24-6.15)

^1^ Only elementary school students were included in the analysis (n = 586).

^2^ Elementary and high school students were included in the analysis (n = 880).

^3^ Binary logistic regression model, adjusted for all included variables.

^4^ Not included in the analysis for elementary school students due to small sample size.

## Discussion

### Main finding of this study

Our results indicated a strong association between cigarette experimentation and alcohol use among elementary and high school students in Crete, Greece in 2007. Moreover, sibling and parental alcohol use, as also having a smoker in the immediate family was independently associated with tobacco experimentation among high school students, thus stressing the important role of the Greek family in influencing substance use.

Indeed, it is common knowledge that parental substance use may modify adolescence substance use [12,13]. Parental smoking has been identified not only as a predictive factor of transitions from never smoking to trying smoking but also from monthly smoking to daily smoking [14-16]. School students in our study who had a smoker in the immediate family were more likely to have experimented with cigarettes than peers without a smoker in the immediate family. Rachiotis et al. also reported that Greek adolescents that have both parents current smokers were twice more likely to be current smokers themselves in comparison to adolescents with non-smoking parents [17]. While Arvanitidou et al., in 2007 also had reported that Greek youth that were smokers were also more likely to be current alcohol drinkers too [10].

The finding that the elementary child’s gender was not found to be associated with cigarette experimentation is in accordance with the Greek 2005 GYTS results, and with other studies that indicate no differentiation by gender [6,7] Smoking prevalence among junior high school students in Greece was estimated in 2005 GYTS at 16.2%, very close to the 17.6% assessed through our study in 2007. Both our study, and the 2005 GYTS data are in contrast with older studies among high-school students conducted in Greece where the smoking rates were found to be higher in males [6-10], indicating a possible change in the paradigm of tobacco use among youth.

Previous research in Greece has identified similar factors to be associated with current smoking such as having a brother or sister that smokes or having more than three friends who actively smoke [18]. Cross sectional research performed within a number of Balkan states and the UK also indicated that substance use by peers and siblings is generally a strong correlate of substance use by adolescents, similar to our findings where alcohol and tobacco use by siblings were determinants of alcohol and tobacco use by the adolescents [8]. While tobacco and alcohol use are associated, they might not act on their own, as other factors may also influence substance abuse, with adolescents who report physical or sexual abuse, violence within the family, stressful life events, or moderate to high depressive symptoms more likely to report regular smoking and regular drinking [19].

Our results, based on 2007 data, indicated that 5% of elementary school students under the age of 10 had already experimented with tobacco and 15% had already experimented with alcohol, which is a cause of concern. So as to combat these habits one of the main approaches for the management of tobacco and alcohol use is a ‘risk-focused approach’ [20]. This approach attempts to eliminate risk factors and in the same time increase the effect of potential protective factors. In that direction, familial attitudes are considered extremely important, as parental modeling is a factor that should be utilized in order to inhibit tobacco and alcohol use during adolescence [21].

### Limitations of this study

While the sample size did allow us to investigate the interrelations between tobacco use and alcohol consumption among elementary and high school students, some analyses are represented with wide confidence intervals, due to the fact that some responses per cell were small. Furthermore, the cross-sectional design of the study and the convenience sample used may not allow for generalizability or the indication of a causal association. As the study was based on a questionnaire a possible recall bias cannot be excluded. These study results could be prone to respondent bias, by which adolescents that smoke or consume a large alcohol in large amount may have opted not to participate in the survey or misreported their consumption. Other known risk factors associated with smoking or alcohol use such as having smoking friends, the amount of pocket money or the parents level of education, history of abuse, violence within the family, depressive symptoms and stressful life events, were not examined [22].

## Conclusions

The current study indicates and association between cigarette experimentation and alcohol consumption in 2007 among youth in Crete, Greece. Preventing adolescents’ experimentation with tobacco is multifactorial including, intervention in entire family, restricting access to and visibility of tobacco products, educating as to the related health effects and disrupting the line of influence of industry actions [23,24].

Understanding the factors associated with substance use and their co-occurrence during adolescence is crucial in health promotion and disease prevention and the above findings stress the need for school-based smoking prevention programs which can be effective in preventing adolescent smoking [25]. Further interventions towards family habits are also considered essential as these may play a key role in adolescent decision making.

## Conflict of interests

The authors have no conflict of interest to declare.

## Authors’ contributions

Authors IT, CIV had the main role in data analysis, interpretation and manuscript preparation, authors IB, EK, EV and MK participated in data collection and interpretation while authors NS and NT were responsible for study design, organization, and participated in data interpretation and manuscript preparation. All authors read and approved the final manuscript.

## Funding

This work was supported by the framework of the Greek INTEREG IIIA to Prof N. Tzanakis.
